# Demand and supply of invasive and noninvasive ventilators at the peak of the COVID‐19 outbreak in Okinawa

**DOI:** 10.1002/jgf2.336

**Published:** 2020-05-26

**Authors:** Toshikazu Kuniya, Yasunori Nakama, Yasuharu Tokuda

**Affiliations:** ^1^ Graduate School of System Informatics Kobe University Kobe Japan; ^2^ Department of Intensive Care Medicine Tomishiro Chuo Hospital Okinawa Japan; ^3^ Muribushi Okinawa Center for Teaching Hospitals Okinawa Japan

**Keywords:** COVID‐19, mechanical ventilation, noninvasive ventilation, respiratory failure, SARS‐CoV‐2

## Abstract

While Okinawa has been facing outbreak of the coronavirus disease 2019 (COVID‐19) pandemic, healthcare collapse should be prevented by sufficient supply of ventilators for caring the rapidly growing number of critically ill patients with COVID‐19. We estimated the number of invasive and noninvasive ventilators that would be required in Okinawa at the peak of the COVID‐19 outbreak based on recent data of COVID‐19 cases in Okinawa and data on the proportion of patients with COVID‐19 in the ICU requiring ventilation. Based on our results using the current supply of all ventilators, demand for ventilators could be prepared for patients with COVID‐19 who would require it and demand for noninvasive ventilators could also be prepared for those with COVID‐19 who would require it. The higher supply over the demand would be achieved by flattening the epidemic curve by implementing public health interventions to delay and suppress the epidemic peak in Okinawa.

## Graphical abstract

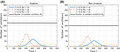

## 1 INTRODUCTION

Hospitals in Okinawa have been facing outbreak of the coronavirus disease 2019 (COVID‐19) because of its pandemic spread.^1^ Although the outbreak has still been in the early phase in Okinawa, it would become serious factor related to healthcare collapse to have insufficient supply of intensive care unit beds and ventilators to care the rapidly growing number of critically ill patients with COVID‐19.^2^ Okinawa is a group of small remote islands having no industry that could rapidly produce ventilators^3^; it may require several days of shipping time for importing ventilators. Thus, we estimated the number of ventilators and noninvasive ventilators that would be required in Okinawa at the peak of the COVID‐19 outbreak to address the intensive care preparation. Our estimates are based on recent data of COVID‐19 cases in Okinawa and data on the proportion of patients with COVID‐19 in the ICU requiring ventilation.

## 2 METHODS

### 2.1 Model setting

We used the well‐known SEIR epidemic model.^4^ The meaning and value of each parameter are shown in Table 1. We regard *t* = 0 as of February 14, 2020, which is the day when the first COVID‐19 case was confirmed in Okinawa.^13^ Thus, *Y*(0) = 1, and hence, *I*(0) = *Y*(0)/(*pN*) ≈ 8.62 ×10^−6^. For simplicity, we assume that *E*(0) = *R*(0) = 0, and thus, *S*(0) = 1 – *I*(0) ≈ 1. The basic reproduction number *R*_0_ ^14^ is calculated as *R*_0_ = *β*/*γ ≈ β*S(0)/*γ*.

**Table 1:** Meaning and value of each parameter used in the model simulation for Okinawa

Symbol	Description	Unit	Value	Reference
*t*	Time	day	0‐365	Assumed
*S*(*t*)	Proportion of susceptible population	‐	0‐1	Estimated
*E*(*t*)	Proportion of exposed population	‐	0‐1	Estimated
*I*(*t*)	Proportion of infective population	‐	0‐1	Estimated
*R*(*t*)	Proportion of removed population	‐	0‐1	Estimated
*Y*(*t*)	Number of confirmed patients	person	*pI*(*t*)*N*	Estimated
*V*_1_(*t*)	Number of required invasive ventilators	machine	*r*_1_ *qY*(*t*)	Estimated
*V*_2_(*t*)	Number of required noninvasive ventilators	machine	*r*_2_ *qY*(*t*)	Estimated
*β*	Infection rate	‐	0.14	Estimated
*R*_0_	Basic reproduction number	person	1.4	Estimated
N	Total population	person	1.45 × 10^6^	^5^
1/*ε*	Average incubation period	day	5	6, 7, 8
1/*γ*	Average infection period	day	10	6, 9
*p*	Identification rate	‐	0.08	^a^
*q*	Proportion of patients possible requiring ventilation	‐	0.05	^10^
*r*_1_	Proportion of requiring invasive ventilation	‐	0.39	^11^
*r*_2_	Proportion of requiring noninvasive ventilation	‐	0.31	^11^
*M*_1_	Number of available invasive ventilators	machine	225	^12^
*M*_2_	Number of available noninvasive ventilators	machine	200	^b^

^a^ Based on diagnostic bias indicated by the government expert committee member (https://www.m3.com/news/iryoishin/761816)

^b^ Personal communication with South West Medical Intelligence, Urasoe City, Okinawa, Japan (http://www.swm‐i.co.jp/company/).

### 2.2 Parameter estimation

**Figure 1:**
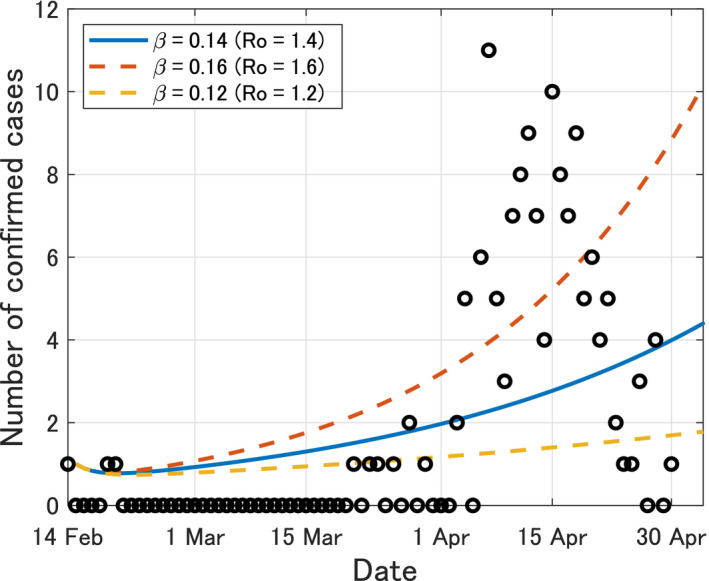
Time variation of the calculated number *Y*(*t*) of confirmed patients in Okinawa from *t* = 0 (February 14, 2020) to *t* = 76 (April 30, 2020). Black circles imply the actual number of confirmed cases^13^

To estimate the infection rate *β*, we apply the least‐squares‐based method^4^ to the data of the daily confirmed cases of COVID‐19 in Okinawa from February 14 to April 30, 2020. As a result, we obtain the estimated value *β = *0.14 (95% CI, 0.12‐0.16), and thus, *R*_0_ = *β*/*γ* = 1.4 (95% CI, 1.2‐1.6). The fitted curves of *Y*(*t*) from *t* = 0 (February 14, 2020) to *t* = 76 (April 30, 2020) are shown in Figure 1.

To estimate the number of required ventilators for COVID‐19 in Okinawa, we assume that the proportion of patients possibly requiring ventilation is *q* = 0.05^10^ and the proportion of those requiring invasive (resp. noninvasive) ventilation is *r*_1_ = 0.39 (resp. *r*_2_ = 0.31).^11^ Thus, the number of required invasive (resp. noninvasive) ventilators can be calculated by multiplying *r*_1_ *q* (resp. *r*_2_ *q*) by the number *Y*(*t*) of confirmed patients. That is, *V*_1_(*t*) = *r*_1_ *qY*(*t*) and *V*_2_(*t*) = *r*_2_q*Y*(*t*).

## 3 RESULTS

### 3.1 Epidemic curves

**Figure 2:**
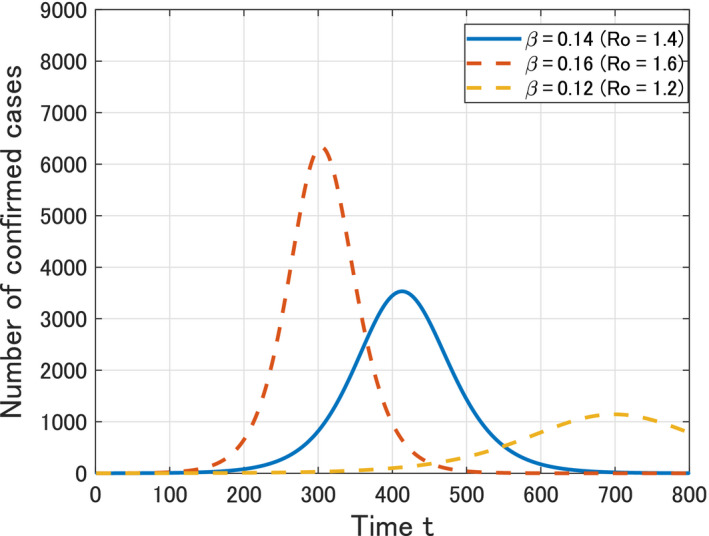
Time variation of the calculated number *Y*(*t*) of confirmed patients in Okinawa from *t* = 0 (February 14, 2020) to *t* = 800 (April 24, 2022)

Using the parameters in Table 1, we obtain the estimation of the epidemic curve of COVID‐19 in Okinawa as shown in Figure 2. As of April 30, 2020, it seems that the epidemic in Okinawa is well controlled and the estimated epidemic curve is sufficiently flattened so that the peak comes late (*t* = 413, ie, April 2, 2021). However, if it goes to the worse scenario (*β* = 0.16 and *R*_0_ = 1.6), then the peak would come within this year (*t = *304, ie, October 14, 2020) and the number of confirmed cases at the peak (≈6334) would become about 1.8 times larger than that for the baseline scenario (≈3532).

### 3.2 Number of required ventilators

**Figure 3:**
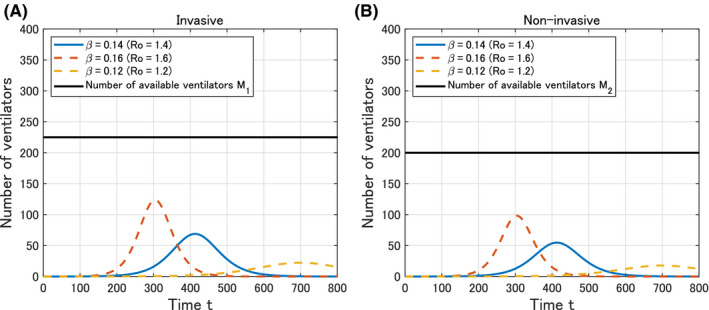
Time variation of the calculated number *V*_1_(*t*) and *V*_2_(*t*) of required invasive and noninvasive ventilators in Okinawa from *t* = 0 (February 14, 2020) to *t* = 800 (April 24, 2022)

We obtain the estimation of the number of required ventilators for COVID‐19 in Okinawa as shown in Figure 3. There is a margin of available ventilators even at the peak in the worse scenario (*β = *0.16 and *R*_0_ = 1.6).

## 4 DISCUSSION

In Okinawa, the number of a ventilator could cover patients with COVID‐19 who would require it, while that of a noninvasive ventilator could also be available for those with COVID‐19 who would require it. Considering that there should be other patients without COVID‐19 requiring invasive or noninvasive ventilations, our estimates should address the lowest preparatory number for ventilator requirement. Reasons for the higher supply over the demand would be that urgent action in Okinawa has been conducted to meet the demand for ventilators. Flattening the epidemic curve was conducted by swiftly and decisively implementing public health interventions to delay and suppress the epidemic peak in Okinawa.^15^

Availability of a noninvasive ventilator in Okinawa in our estimate makes Okinawa better prepared for the outbreak. Although a noninvasive ventilator had been less used for patients with COVID‐19 to avoid possible risk of aerosol infection to healthcare providers, a recent review recommended it could be used safely at the use of complete infection preventive measures and might be preferably used to reduce the high complication rate related to the use of invasive ventilators and the infection risk associated with them.^16^

There are several limitations in our estimates. First, since the number of diagnosis of infected patients is likely to underestimate actual number of patients because of policy for restricting test only for patients with severe disease in Japan, we may face the greater number of patients with both mild and severe diseases.^17^ Second, the pandemic might subside from specific climate conditions in Okinawa such as higher temperature and humidity along with ultraviolet radiation exposure in summer.^18^ Third, the coinfection of COVID‐19 and influenza in winter may lead to higher incidence of severity, which may increase the demand.^19^

In conclusion, although it would be disastrous to wait and see until the possible supply shortage of life‐saving ventilators, the requirement of ventilators would not exceed its availability during the period of expected peak based on our estimate about the highest demand scenario. Urgent actions conducted in Okinawa would save lives and avoid devastating rationing that would require physicians not to be able to allocate ventilators to some of patients with COVID‐19.

## CONFLICT OF INTEREST

The authors have stated explicitly that there are no conflicts of interest in connection with this article.
